# Remission of Persistent Hypothyroidism Following Subacute Thyroiditis After Discontinuation of Thyroxine: A 9‐Year Retrospective Study

**DOI:** 10.1155/ije/8820514

**Published:** 2026-01-07

**Authors:** Lei Yang, Min Mao, Lian Duan

**Affiliations:** ^1^ Department of Breast and Thyroid Surgery, The Third Affiliated Hospital of Chongqing Medical University, Chongqing, China, cqmu.edu.cn; ^2^ Department of Hematology, The First Affiliated Hospital of Chongqing Medical University, Chongqing, China, cqmu.edu.cn; ^3^ Department of Endocrinology, The Third Affiliated Hospital of Chongqing Medical University, Chongqing, China, cqmu.edu.cn

**Keywords:** cohort study, hypothyroidism, prognosis, subacute thyroiditis, withdrawal

## Abstract

**Objective:**

The long‐term outcomes of discontinuing thyroxine replacement therapy in patients with persistent hypothyroidism following subacute thyroiditis are unknown. This study involved an extended follow‐up of a cohort of patients who participated in a clinical trial of prednisone for the treatment of subacute thyroiditis.

**Methods:**

This retrospective cohort study included 52 patients with moderate to severe scores who were hospitalized between August 2013 and December 2014. Patients previously received prednisone for 1 week, followed by nonsteroidal anti‐inflammatory drugs for 1 week, and prednisone was administered for 6 weeks, after which the patients completed follow‐up to Week 24. Thyroid‐stimulating hormone, free triiodothyronine, and free thyroxine levels of the participants were measured 9 years after enrollment.

**Results:**

Of the 52 participants randomly assigned to receive prednisone for 1 or 6 weeks, 50 completed the core trial, and 48 were eligible for extended follow‐up, with a median duration of 8.61 years (IQR 8.29−8.77). Thirty participants were assessed at 9 years, 15 could not be contacted, and three refused follow‐up tests. Among the 30 participants, 28 were euthyroid and 2 had subclinical hypothyroidism at 9 years. The median TSH level was 3.46 mIU/L (IQR 2.12–5.15) at 24 weeks and 2.17 mIU/L (IQR 1.83–3.77) at 9 years (*p* = 0.001). The median FT4 level was 14.27 pmol/L (IQR 12.15–15.72) at 24 weeks and 15.28 pmol/L (IQR 12.53–16.22) at 9 years (*p* = 0.959). Among the three participants diagnosed with persistent hypothyroidism at 24 weeks, one participant was diagnosed with subclinical hypothyroidism without thyroxine replacement therapy at 9 years, and two participants were diagnosed with subclinical hypothyroidism and euthyroidism after gradually withdrawing from thyroxine.

**Conclusion:**

Thyroid function remains stable in patients with persistent hypothyroidism following subacute thyroiditis after careful dose reduction and discontinuation of thyroxine replacement therapy. This finding may have implications for the individualized management of hypothyroidism.

## 1. Introduction

Patients with hypothyroidism generally receive lifelong thyroxine therapy [1]. Discontinuing unnecessary thyroxine replacement therapy could avoid side effects such as increased risk of arrhythmias, angina, osteoporosis, and fractures [2–4]. Among patients with idiopathic or autoimmune hypothyroidism, 37.2% of those who discontinued thyroxine remained euthyroid; unfortunately, 65.8% needed to restart thyroid hormone therapy during the subsequent follow‐up [5]. It is difficult to predict which patients can successfully discontinue thyroxine therapy; therefore, physicians generally continue thyroxine replacement therapy indefinitely.

Subacute thyroiditis (SAT) is a common cause of hypothyroidism [6] and a self‐limiting condition [7]. Hypothyroidism follows SAT, with the incidence of persistent hypothyroidism ranging from 14.3% to 26.8% [8, 9]. The long‐term outcomes are unclear, especially in patients with persistent hypothyroidism. Quintero [10] suggested that thyroxine replacement could be discontinued 12 months after SAT. However, a previous study revealed that more than one‐quarter of patients with SAT had persistent hypothyroidism 3 years after starting treatment [9]. Our previous research suggested that participants could gradually reduce and discontinue thyroxine with an extended follow‐up from 24 weeks (the endpoint of the core trial) to 4 years [11]. It is uncertain whether patients who discontinue thyroxine later need to reinitiate the therapy, as is the case in patients with autoimmune and idiopathic hypothyroidism. Therefore, the follow‐up of trial patients with SAT was extended to 9 years.

## 2. Materials and Methods

We affirm that no AI‐generated content tools based on large language models (e.g., ChatGPT) were used in any part of the preparation of this manuscript.

### 2.1. Study Design and Participants

This extended follow‐up study aimed to ascertain whether patients who discontinued thyroxine replacement therapy remained euthyroid or required reinitiation of thyroid hormone replacement.

The original study was a single‐center, randomized controlled trial involving 52 patients aged 18–70 years who were hospitalized for SAT between August 2013 and December 2014. The patients had moderate to severe SAT scores. SAT severity was scored as described previously [12]. SAT scores ≥ 3 points were defined as moderate‐to‐severe SAT [11]. Patients with moderate‐to‐severe symptoms were randomly assigned to receive either 30 mg/day prednisone for 1 week, followed by 1 week of nonsteroidal anti‐inflammatory drugs, or the conventional 6‐week prednisone therapy. The trial endpoints were assessed at 24 weeks. The primary endpoint was treatment efficacy at the end of the treatment course, and the secondary endpoints included between‐group differences in the incidence of adverse effects and thyroid function at Weeks 6, 12, and 24.

Participants who completed the follow‐up until Week 24 were invited to participate in the observational study with extended follow‐up. These participants were followed up to Year 9. This study was approved by the ethics committee of the university hospital (grant number 2024014), and all participants provided written informed consent.

### 2.2. Procedures

The participants recorded any discomfort during the extended follow‐up period. The required medical assistance for discomfort, regular follow‐up of persistent hypothyroidism, and thyroid hormone dose adjustment for participants were provided by the doctors after the core trial. The follow‐up intervals after 24 weeks will be freely determined by the participants on the basis of their symptoms and the attending physician’s understanding of the condition. Free triiodothyronine (FT3), free thyroxine (FT4), and thyroid‐stimulating hormone (TSH) levels were measured at the 9‐year visit.

### 2.3. Thyroid Function and Autoantibody Testing

The serum levels of FT3, FT4, TSH, and/or thyroid‐binding globulin (TG), antithyroglobulin antibody (TGAb), antithyroid peroxidase antibody (TPOAb), and antithyroid‐stimulating hormone receptor antibody (TRAb) were measured via electrochemiluminescence immunoassays (different local testing systems, such as the Beckman Coulter UniCel DxI 800, Immunoassay System, USA, and the Roche Cobas e601 Electrochemical Luminescence Immunoassay Analyzer, SWIT). The reference ranges provided by the manufacturer were 3.1–6.8 pmol/L for FT3, 11–22 pmol/L for FT4, 0.465–4.68 mIU/L for TSH, 1.4–78 μg/L for TG, 0–115 IU/mL for TGAb, 0–40 IU/mL for TPOAb, and 0–1.75 IU/L for TRAb. Subclinical hypothyroidism (SCH) is characterized by a TSH level between the upper reference limit and 10 mIU/L combined with a normal FT4 level [13]. Overt hypothyroidism (OH) is characterized by elevated TSH levels (> 10 mIU/L) and subnormal FT4 levels [14]. Persistent hypothyroidism was defined as hypothyroidism persisting beyond the 24‐week follow‐up [11].

### 2.4. Statistical Analysis

The Shapiro–Wilk test was used to evaluate the normality of continuous variables presented as the means ± SDs or medians with interquartile ranges (IQRs). Categorical variables are expressed as numbers and percentages. The Wilcoxon test was used to explore the differences between FT4 or TSH levels at follow‐ups at 24 weeks and 9 years. All the statistical analyses were conducted via SPSS (Version 20.0; IBM Corp., Armonk, NY, USA). Two‐tailed *p* values < 0.05 were considered statistically significant.

## 3. Results

### 3.1. The Clinical Characteristics of the Participants in the Core and Extension Trials

**Figure 1:**
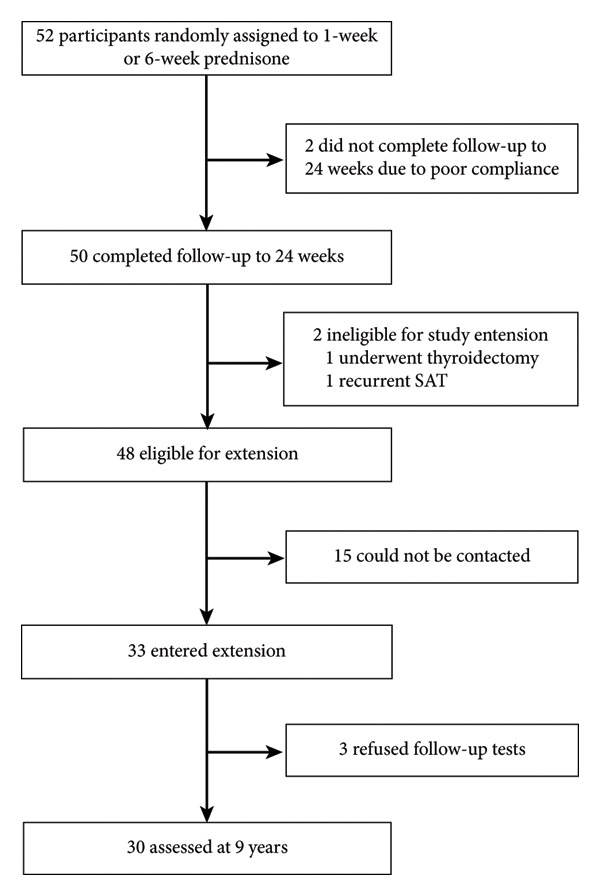
Core trial and extension profile. Abbreviation: SAT, subacute thyroiditis.

Fifty‐two participants were randomly assigned to receive short‐term or 6‐week prednisone treatment in the core trial. A total of 50 participants completed the core trial. One patient in the short‐term group and one in the 6‐week group did not complete the follow‐up until 24 weeks due to poor compliance. The TSH levels exceeded the normal upper limit at 6 weeks and then decreased to the normal range at 12 and 24 weeks, which was the endpoint of a previous study [11]. There were 48 eligible participants for the extension between February 17, 2014, and August 25, 2023. One patient who underwent thyroidectomy for benign thyroid nodules and one with recurrent SAT were excluded. Moreover, 18 patients were excluded because they were lost to follow‐up, including 15 who could not be contacted (multiple phone calls without a response or phone number changes) and three who refused follow‐up tests. At Year 9 of follow‐up, 30 (62.5%) participants were assessed (Figure 1); the median duration of further follow‐up was 8.61 years (IQR 8.29–8.77). The frequency range for monitoring thyroid function during the extended follow‐up period was 1–16 times. The clinical characteristics of the participants in the core and extension trials are presented in Table 1. The last follow‐up visit was on August 25, 2023. None of the participants reported symptoms associated with hypothyroidism during the extended follow‐up period. No thyroxine‐induced hyperthyroidism was detected.

**Table 1:** Clinical characteristics of participants in the core and extension trials.

	Core cohort at trial baseline (*n* = 50)	Extension cohort at trial baseline (*n* = 48)	Extension cohort at extension endpoint (*n* = 30)
Age, years	43.50 (39.75–51.00)	43.50 (40.00–51.00)	53.00 (50.00–61.00)
Gender, female	40 (80%)	38 (79%)	25 (83%)
TPOAb, IU/mL	13.00 (5.00–32.00)	NA	6.11 (0.99–11.03)
TGAb, IU/mL	49.43 (33.63–418.20)	NA	18.55 (0.80–24.51)

*Note:* TGAb, antithyroglobulin antibody; TPOAb, antithyroid peroxidase antibody. Data are median (IQR) and *n* (%).

Abbreviation: NA, not applicable.

### 3.2. Changes in FT4 and TSH Levels at the Extension Trial

**Figure 2:**
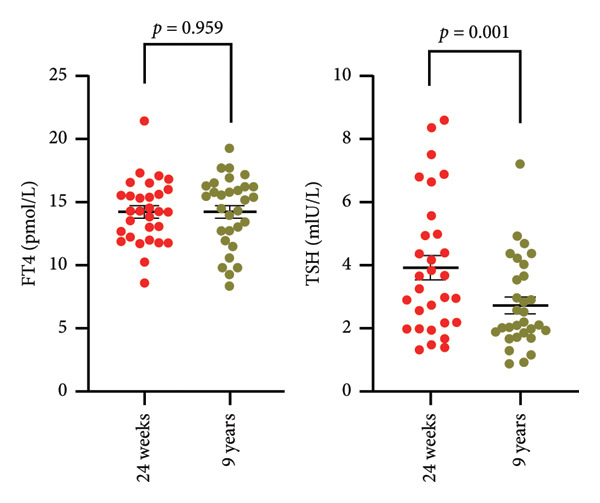
Changes in FT4 and TSH levels at the endpoint of the core and extension trials. Abbreviations: FT4, free thyroxine; TSH, thyroid‐stimulating hormone.

Among the 30 participants, 28 were euthyroid and 2 had SCH (Supporting Table 1). The median value of the TSH level was 3.66 mIU/L (IQR 2.19–5.30) at 24 weeks in the trial and 2.51 mIU/L (IQR 1.87–3.85) at the 9‐year follow‐up (*p* = 0.001). The median FT4 level was 14.24 pmol/L (IQR 12.10–15.57) at 24 weeks of the trial and 14.00 pmol/L (IQR 11.78–15.8) at the 9‐year follow‐up (*p* = 0.959) (Figure 2). The other items, except for FT4 and TSH, of the 30 participants at the 9‐year follow‐up are presented in Supporting Table 2. The prevalence of persistent hypothyroidism at the end of the core trial was 3/50 (6%). Among the three participants diagnosed with persistent hypothyroidism at 24 weeks, one participant in the 6‐week prednisone treatment group was diagnosed with SCH without thyroxine replacement therapy at 9 years. Two participants in the 1‐week prednisone followed by 1‐week nonsteroidal anti‐inflammatory drugs group were diagnosed with SCH and euthyroidism at 9 years and gradually withdrawn from thyroxine after thyroxine replacement therapy for 3 years, 8 months, or 4 years (Supporting Table 3).

### 3.3. Changes in FT4 and TSH Levels in Three Participants With Persistent Hypothyroidism During the 9‐Year Follow‐Up Period

**Figure 3:**
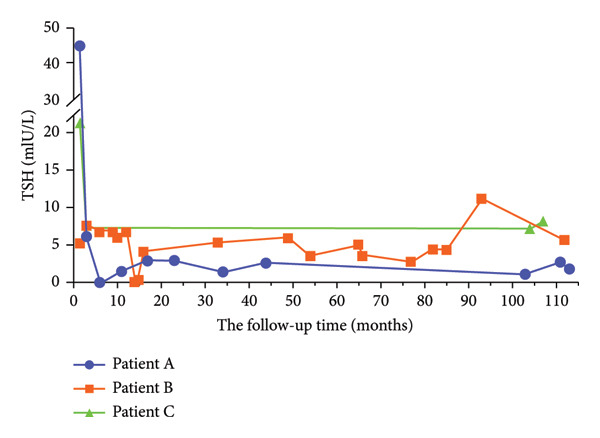
Changes in TSH levels in three patients with persistent hypothyroidism during the 9‐year follow‐up period. Abbreviation: TSH, thyroid‐stimulating hormone.

A 36‐year‐old woman (Patient A) with a TPOAb level of 67 IU/mL (0–9), TGAb level of 121.8 IU/mL (0–4), and TSH level of 44.81 mIU/L (0.56–5.91) at 6 weeks after the onset of SAT was diagnosed with OH. She was treated with gradually increasing doses of thyroxine, starting at 25 μg/day and increasing to 75 μg/day. During the following 3 years and 8 months, her thyroid hormone levels gradually decreased, and thyroxine was eventually withdrawn because her thyroid function and thyroid autoantibodies returned to normal after multiple follow‐up tests. The patient was euthyroid at the time of treatment withdrawal (May 27, 2017). At the 9‐year follow‐up, her TSH and FT4 levels were 1.86 mIU/L (0.56–5.91) and 14.6 pmol/L (7.98–16.02), respectively (Figure 3).

A 28‐year‐old woman (Patient B) with a TPOAb level of 9 IU/mL (0–9), TGAb level of 43.6 IU/mL (0–4), FT4 level of 12.7 pmol/L (7.98–16.02), and TSH level of 6.91 mIU/L (0.56–5.91) at 24 weeks after onset was diagnosed with SCH. She was treated with thyroxine, starting at 25 μg/day and increasing to 50 μg/day owing to an unexpected pregnancy. She was treated with thyroxine during pregnancy, which was gradually reduced and continued for more than three years after delivery in December 2014. Treatment was eventually discontinued in April 2018 after the patient took thyroxine for four years. The patient experienced SCH after withdrawal and maintained this status for the following 5 years. At the 9‐year follow‐up, her TSH level was 5.75 (0.56–5.91) mIU/L and her FT4 level was 11.6 pmol/L (7.98–16.02) (Figure 3).

A 48‐year‐old woman (Patient C) with TPOAb, TGAb, FT4, and TSH levels of 23 IU/mL (0–9), 22.5 IU/mL (0–4), 14.2 pmol/L (7.98–16.02), and 7.54 mIU/L (0.56–5.91), respectively, at 24 weeks was diagnosed with SCH and did not receive thyroxine replacement therapy. At the 9‐year follow‐up, her TSH level was 7.24 mIU/L (0.56–5.91), and her FT4 level was 15.4 pmol/L (7.98–16.02) (Figure 3).

## 4. Discussion

Hypothyroidism is a common condition that is often irreversible and requires lifelong thyroxine replacement therapy [15]. No recommendations are available regarding whether or when to stop thyroxine replacement therapy, and the current guidelines do not recommend continuous evaluation of the need for the therapy [13, 14, 16]. Among patients with idiopathic or autoimmune hypothyroidism who discontinued thyroxine, 37.2% remained euthyroid; however, 65.8% restarted thyroxine during the subsequent follow‐up from 3 weeks to a median of 5 years [5]. It is unclear whether participants with hypothyroidism following SAT who later discontinued thyroxine needed to reinitiate thyroid hormone replacement, as was the case in participants with autoimmune and idiopathic hypothyroidism. Our results suggest that in patients with persistent hypothyroidism after SAT, thyroid function remains stable after careful dose reduction and discontinuation of thyroxine replacement therapy.

The long‐term outcomes of persistent hypothyroidism after SAT are of interest. Few studies have reported the long‐term outcomes of SAT, particularly in participants with persistent hypothyroidism. Cohort studies of patients with SAT at 7 and 30 years of follow‐up have reported a prevalence of persistent hypothyroidism of 14.3% and 25%, respectively [8, 9]. We found the following: (1) During the follow‐up period of the core trial, 61% of patients experienced hypothyroidism, including OH (28%) and SCH (33%); (2) At the end of the trial (24 weeks), three patients (6%) were diagnosed with persistent hypothyroidism [11], similar to the 5%–15% prevalence of persistent hypothyroidism reported in the guidelines for SAT [17]. However, even 5 years after withdrawal, the thyroid function of the cohort remained stable. Although the median TSH level at the 9‐year follow‐up differed significantly from that at the 24‐week follow‐up, both the TSH and FT4 levels were within the normal reference ranges, so the difference in TSH levels was not clinically significant. Current guidelines recommend thyroid hormone replacement for adults with TSH levels > 10 mIU/L [13]; therefore, patients with persistent hypothyroidism did not restart thyroxine in our study. These results indicate that persistent hypothyroidism may not be permanent in this population.

When primary hypothyroidism is related to a specific cause, such as SAT, it is less likely to be persistent [18], which means that withdrawal may be feasible. The biggest characteristic of SAT is self‐limiting, and thyroid tissue structure can return to normal after the disease. This is an important reason for the majority of participants to return to normal function without deterioration. There are clinical guidelines on when to initiate thyroxine replacement therapy [13, 14, 16], but very limited research has been conducted on when to discontinue thyroxine replacement therapy. A shorter duration of thyroxine therapy and a lower thyroxine dose at the time of tapering are predictable factors for successful thyroxine tapering [19]. On the basis of our cohort and other researchers, the following three scenarios can lead to the discontinuation of thyroxine: (1) Patients with TSH within the normal reference range for several years following SAT should be offered a trial of thyroxine withdrawal if they have negative antibodies. (2) Patients with “mild” hypothyroidism with low doses of thyroxine (25–50 μg/day) may be given a thyroxine withdrawal trial. (3) A 3‐week withdrawal trial should be considered, considering that the mean half‐life of L‐thyroxine is approximately 7 days [20]. Thyrotropin and thyroxine levels should be measured after this period. If normal, the patient can be assumed to be euthyroid. Elevated TSH and low thyroxine levels indicate hypothyroidism, and hormone therapy should be resumed [18]. After continuous observation for 3–6 months after withdrawal, if thyroid function remains stable, the interval between reevaluations can be gradually extended annually. The above three rules can help physicians determine which patients can attempt thyroxine withdrawal and the duration of trial withdrawal, and they can alleviate patients′ anxiety about gradually reducing and stopping thyroxine if their thyroid function is normal.

Therefore, it is necessary to explore the predictors of persistent hypothyroidism. Female sex, a high cumulative dose of prednisolone, and a greater reduction in thyroid volume within 1 month of the episode were associated with a greater risk of persistent hypothyroidism [9, 21]. The limited participants in this study all had severity scores ≥ 3 at initial diagnosis, which is defined as moderate or severe symptoms, and none had OH at the 9‐year follow‐up, suggesting that symptom severity at diagnosis may not be a predictor of persistent hypothyroidism. Although the difference was not statistically significant, the risk of developing hypothyroidism was greater in TPOAb‐positive patients (38.5%) than in TPOAb‐negative patients (25.4%) [9]. Several studies have shown that TPOAb positivity is the main risk factor for persistent hypothyroidism after SAT [9, 22, 23]. However, when autoantibodies become negative after a previous positive result, that positivity could be due to the antigen release during the destructive process and in this case the positivity is not anymore a risk factor to permanent hypothyroidism. TPOAb positivity is closely related to autoimmune thyroiditis [24]. Autoimmune thyroiditis initially occurs, and concurrent SAT can enhance autoimmune processes that eventually result in hypothyroidism [25]. In our cohort, most participants maintained low concentrations of TPOAb and TGAb at diagnosis and were euthyroid at 9 years.

Among the three participants who were diagnosed with persistent hypothyroidism at 24 weeks, Patient A was positive for autoimmune antibodies at the initial diagnosis, and the level of autoimmune antibodies gradually decreased to within the normal range in subsequent re‐evaluations. Patients B and C were negative for autoimmune antibodies. These findings suggest that autoimmune thyroid antibody positivity rather than disease severity may be an important predictor of hypothyroidism after SAT. Pregnancy without complete recovery from thyroid follicular damage may be related to persistently unstable thyroid function. Careful identification of the etiology of primary hypothyroidism, such as autoimmune thyroiditis and SAT, at the initial visit is important for assessing the need for continuous thyroxine replacement therapy.

To the best of our knowledge, outcomes after the discontinuation of thyroid hormone therapy in patients with persistent hypothyroidism following SAT have not been reported previously. In this study, under the management of the above discontinuation rules, thyroxine was withdrawn approximately 4–9 years after diagnosis and permanent hypothyroidism was reversed. The 24‐week or 1‐year [9] threshold used to define persistent hypothyroidism may not be absolute, and follow‐up may be appropriately extended to approximately 4 years in patients with SAT. Continuous reassessment of thyroxine therapy and determination of the appropriate dose should be considered for optimal treatment.

This study had several limitations. There may be selection bias due to the high number of participants who could not be contacted after the COVID‐19 epidemic and the unwillingness of some participants to undergo follow‐up screening due to a lack of symptoms. The small sample size, nonprospective, nonrandomized, noncontrolled design, and lack of thyroid ultrasound evaluation may have limited the generalizability of our results. Finally, we did not account for the impacts of different glucocorticoid treatment courses, gender, and age between the two groups on the prognosis of persistent hypothyroidism. However, these findings serve as a reference for investigating thyroxine withdrawal in patients with persistent hypothyroidism following SAT.

## 5. Conclusion

Hypothyroidism due to SAT can be reversed a few years after diagnosis without the need for thyroxine replacement therapy. This finding may have implications for the individualized management of hypothyroidism. The clinical guiding value of the research is limited to some extent by small sample size; however, the results of the study are valuable for understanding the long‐term prognosis of SAT. Further studies with expanded sample sizes are warranted to validate the clinical significance of these preliminary findings.

**Nomenclature**
FT3: Free triiodothyronine
FT4: Free thyroxine
IQR: Interquartile range
OH: Overt hypothyroidism
TSH: Thyroid‐stimulating hormone
TG: Thyroid‐binding globulin
TGAb: Antithyroglobulin antibody
TPOAb: Antithyroid peroxidase antibody
TRAb: Antithyroid‐stimulating hormone receptor antibody
SAT: Subacute thyroiditis
SCH: Subclinical hypothyroidism

## Disclosure

All authors reviewed and approved the final manuscript.

## Conflicts of Interest

The authors declare no conflicts of interest.

## Author Contributions

Lian Duan designed the study, supervised the data collection process, checked the final analysis results, and revised the manuscript. Lei Yang analyzed data and participated in the drawing. Min Mao collected data, completed the follow‐up, and participated in drafting the initial manuscript. Lei Yang and Min Mao contributed equally and are considered co‐first authors.

## Funding

This study was funded by Chongqing medical scientific research project (Joint project of Chongqing Health Commission and Science and Technology Bureau) (2022GDRC016) and High‐level Medical Reserved Personnel Training Project of Chongqing (CQSZQNYXGDRC201829).

## Supporting Information

Additional supporting information can be found online in the Supporting Information section.

## Supporting information


**Supporting Information 1** Supporting Table 1: The FT4 and TSH levels of 30 participants at the 24‐week and 9‐year follow‐ups.

**Supporting Information 2** Supporting Table 2: Other items except for FT4 and TSH in thyroid function of 30 participants at the 9‐year follow‐up.

**Supporting Information 3** Supporting Table 3: Changes in TGAb, TPOAb, and TSH levels during a 9‐year follow‐up in three participants with persistent hypothyroidism.

## Data Availability

The datasets generated for this study can be obtained upon reasonable request by email to the corresponding author.
