# Supplementary material for: Remission of Persistent Hypothyroidism Following Subacute Thyroiditis After Discontinuation of Thyroxine: A 9‐Year Retrospective Study
Source: Int J Endocrinol. 2026 Jan 7;2026:8820514. doi: 10.1155/ije/8820514 (PMC12775677; doi:10.1155/ije/8820514)
Supplement: Supplementary file 1 — Supporting Information 1 Supporting Table 1: The FT4 and TSH levels of 30 participants at the 24‐week and 9‐year follow‐ups. [file IJE-2026-8820514-s002.docx]

Supplementary Table 1 The FT4 and TSH levels of 30 participants at the 24-week and 9-year

| No. | FT4(pmol/L) | | TSH(mIU/L) | |
| --- | --- | --- | --- | --- |
|  | 24Week | 9 Year | 24Week | 9 Year |
| 1* | 15.50 | 14.56 | 1.38 | 1.86 |
| 2 | 13.10 | 15.89 | 3.69 | 4.70 |
| 3 | 15.30 | 16.26 | 6.83 | 4.23 |
| 4* | 12.70 | 11.46 | 6.91 | 4.93 |
| 5 | 8.60 | 15.76 | 3.01 | 2.92 |
| 6 | 11.80 | 15.83 | 4.40 | 4.40 |
| 7 | 15.40 | 13.40 | 1.98 | 1.68 |
| 8 | 21.50 | 15.43 | 3.26 | 3.68 |
| 9 | 15.60 | 12.73 | 3.66 | 0.90 |
| 10 | 13.00 | 12.72 | 5.58 | 3.54 |
| 11 | 13.50 | 16.90 | 8.37 | 2.59 |
| 12 | 11.70 | 10.60 | 4.18 | 4.38 |
| 13 | 13.80 | 13.00 | 1.31 | 1.89 |
| 14 | 11.80 | 9.23 | 2.75 | 2.09 |
| 15 | 16.00 | 15.17 | 2.58 | 1.95 |
| 16 | 10.30 | 16.21 | 4.96 | 2.12 |
| 17 | 14.60 | 8.35 | 3.84 | 2.83 |
| 18 | 15.50 | 9.83 | 2.97 | 2.21 |
| 19 | 14.30 | 17.74 | 2.17 | 1.73 |
| 20* | 14.20 | 15.39 | 7.54 | 7.24 |
| 21 | 17.30 | 17.70 | 1.96 | 1.16 |
| 22 | 12.20 | 15.58 | 5.01 | 4.03 |
| 23 | 16.50 | 14.28 | 1.98 | 1.31 |
| 24 | 16.60 | 16.20 | 4.39 | 2.02 |
| 25 | 17.10 | 14.00 | 2.21 | 2.05 |
| 26 | 16.80 | 16.57 | 2.92 | 2.98 |
| 27 | 14.20 | 11.95 | 8.61 | 2.51 |
| 28 | 14.30 | 19.30 | 1.50 | 0.94 |
| 29 | 11.90 | 17.20 | 1.68 | 1.99 |
| 30 | 12.00 | 9.79 | 6.67 | 1.68 |

Abbreviations: FT4, free thyroxine; TSH, thyroid-stimulating hormone. * The participant with permanent hypothyroidism
