# Supplementary material for: Remission of Persistent Hypothyroidism Following Subacute Thyroiditis After Discontinuation of Thyroxine: A 9‐Year Retrospective Study
Source: Int J Endocrinol. 2026 Jan 7;2026:8820514. doi: 10.1155/ije/8820514 (PMC12775677; doi:10.1155/ije/8820514)
Supplement: Supplementary file 2 — Supporting Information 2 Supporting Table 2: Other items except for FT4 and TSH in thyroid function of 30 participants at the 9‐year follow‐up. [file IJE-2026-8820514-s003.docx]

Supplementary Table 2 Other items except for FT4 and TSH in thyroid function of 30 participants at the 9-year

| No. | FT3(pmol/L) | TG(µg/L) | TGAb(IU/mL) | TPOAb(IU/mL) | TRAb(IU/L) |
| --- | --- | --- | --- | --- | --- |
| 1* | 4.62 | - | 0.67 | 0.96 | 0.916 |
| 2 | 6.41 | 5.42 | 20.97 | 14.13 | <0.8 |
| 3 | 3.49 | 5.45 | <10 | <0.25 | - |
| 4* | 3.2 | 11.7 | 0.1 | 2.62 | 0.25 |
| 5 | 4.8 | 3.53 | 23.41 | 9.45 | 1.43 |
| 6 | 4.42 | - | <10 | 0.45 | - |
| 7 | 2.9 | 3.9 | 72 | 5.71 | - |
| 8 | 5.14 | - | 25.6 | 59.5 | - |
| 9 | 4.51 | 11.13 | - | - | - |
| 10 | 4.66 | - | 21.32 | 7.78 | - |
| 11 | 3.14 | - | 0.5 | 15.2 | - |
| 12 | 4.06 | - | <20 | <10 | - |
| 13 | 3.3 | - | 0.1 | 6.5 | - |
| 14 | 2.53 | - | 1 | 1 | - |
| 15 | 5.27 | - | 64.7 | 30.2 | - |
| 16 | 4.86 | - | 0.7 | 0.4 | - |
| 17 | 3.48 | - | - | - | - |
| 18 | 5.43 | - | 0.4 | 3.8 | - |
| 19 | 3.54 | - | < 0.9 | 0.36 | - |
| 20* | 3.91 | 47.59 | 18.55 | - | - |
| 21 | 5.67 | - | 19.92 | 24.81 | 2.72 |
| 22 | 4.86 | - | 16.54 | 1.14 | - |
| 23 | 5.28 | - | - | - | - |
| 24 | 3.53 | - | 139.3 | 9.72 | 0.82 |
| 25 | 4.26 | - | 249.5 | 8.84 | - |
| 26 | 4.99 | - | - | - | - |
| 27 | 4.66 | - | - | - | - |
| 28 | 4.2 | 1.55 | 19.74 | < 3 | - |
| 29 | 5.03 | - | 139.34 | - | 0.8 |
| 30 | 6.26 | 3.47 | 1 | - | 1.42 |

Abbreviations: FT3, free triiodothyronine; TG, thyroid-binding globulin; TGAb, antithyroglobulin antibody; TPOAb, antithyroid peroxidase antibody; TRAb, antithyroid-stimulating hormone receptor antibody; –, Not available.

* The participant with permanent hypothyroidism
