# Supplementary material for: Remission of Persistent Hypothyroidism Following Subacute Thyroiditis After Discontinuation of Thyroxine: A 9‐Year Retrospective Study
Source: Int J Endocrinol. 2026 Jan 7;2026:8820514. doi: 10.1155/ije/8820514 (PMC12775677; doi:10.1155/ije/8820514)
Supplement: Supplementary file 3 — Supporting Information 3 Supporting Table 3: Changes in TGAb, TPOAb, and TSH levels during a 9‐year follow‐up in three participants with persistent hypothyroidism. [file IJE-2026-8820514-s001.docx]

Supplementary Table 3 Changes of TGAb, TPOAb, and TSH levels during a 9-year follow-up in three participants with persistent hypothyroidism

| Time | Patient A | | | | Patient B | | | | Patient C | | | |
| --- | --- | --- | --- | --- | --- | --- | --- | --- | --- | --- | --- | --- |
|  | TGAb  (IU/mL) | TPOAb  (IU/mL) | TSH  (mIU/L) | Thyroxine  (ug/d) | TGAb  (IU/mL) | TPOAb  (IU/mL) | TSH  (mIU/L) | Thyroxine  (ug/d) | TGAb  (IU/mL) | TPOAb  (IU/mL) | TSH  (mIU/L) | Thyroxine  (ug/d) |
| Baseline | 128.1 | 67 | 0.005 |  | 43.6 | 9 | 0.005 |  | 22.5 | 23 | 0.024 |  |
| 1.5M | - | - | 44.81 | 25 | - | - | 5.25 | - | - | - | 21.3 | - |
| 3M | - | - | 6.17 | 75 | - | - | 7.62 | - | - | - | 7.54 | - |
| 6M | - | - | 0.13 | 75 | - | - | 6.91 | 12.5 | - | - | - | - |
| 9M | - | - | - | - | 16 | 5 | 6.89 | 50 | - | - | - | - |
| 10M | - | - | - | - | - | - | 6.008 | - | - | - | - | - |
| 11M | - | - | 1.55 | 50 | - | - | - | - | - | - | - | - |
| 12M | - | - | - | - | 15.7 | 26 | 7.01 | - | - | - | - | - |
| 14M | - | - | - | - | - | - | 0.143 | - | - | - | - | - |
| 15M | - | - | - | - | - | - | 0.384 | - | - | - | - | - |
| 16M | - | - | - | - | - | - | 4.1 | - | - | - | - | - |
| 17M | 27.2 | < 5 | 2.91 | 25 | - | - | - | - | - | - | - | - |
| 23M | - | - | 2.96 | - | - | - | - | - | - | - | - | - |
| 33M | - | - | - |  | - | - | 5.37 | - | - | - | - | - |
| 34M | 15.9 | 16 | 1.4 | - | - | - | - | - | - | - | - | - |
| 44M | < 10 | 19 | 2.61 | withdrawal | - | - | - | - | - | - | - | - |
| 49M | - | - | - | - | 22.81 | 9.7 | 5.95 | withdrawal | - | - | - | - |
| 54M | - | - | - | - | 46.85 | 35.78 | 3.55 | - | - | - | - | - |
| 65M | - | - | - | - | < 10 | 13.81 | 5.04 | - | - | - | - | - |
| 66M | - | - | - | - | - | - | 3.63 | - | - | - | - | - |
| 77M | - | - | - | - | 10.3 | 6.23 | 2.817 | - | - | - | - | - |
| 82M | - | - | - | - | < 10 | < 3 | 4.496 | - | - | - | - | - |
| 85M | - | - | - | - | 0 | 8.3 | 4.391 | - | - | - | - | - |
| 93M | - | - | - | - | 0.1 | 6.1 | 11.213 | - | - | - | - | - |
| 103M | - | - | 1.148 | - | - | - | - | - | - | - | - | - |
| 104M | - | - | - | - | - | - | - | - | 18.55 | - | 7.24 | - |
| 107M | - | - | - | - | - | - | - | - | - | - | - | - |
| 111M | 0.3 | 0.4 | 2.748 | - | - | - | - | - | - | - | - | - |
| 112M | - | - | - | - | - | 2.62 | 5.75 | - | - | - | - | - |
| 113M | - | - | 1.858 | - | - | - | - | - | - | - | - | - |

Abbreviations: M, Month; TGAb, antithyroglobulin antibody; TPOAb, antithyroid peroxidase antibody; TSH, thyroid-stimulating hormone; –, Not available.
